# Commercial Biocontrol Agents Reveal Contrasting Comportments Against Two Mycotoxigenic Fungi in Cereals: *Fusarium Graminearum* and *Fusarium Verticillioides*

**DOI:** 10.3390/toxins12030152

**Published:** 2020-02-29

**Authors:** Lucile Pellan, Noël Durand, Véronique Martinez, Angélique Fontana, Sabine Schorr-Galindo, Caroline Strub

**Affiliations:** 1 Qualisud, Univ Montpellier, CIRAD, Montpellier SupAgro, Univ d’Avignon, Univ de La Réunion, Montpellier, France; noel.durand@cirad.fr (N.D.); veronique.martinez@umontpellier.fr (V.M.); angelique.fontana@umontpellier.fr (A.F.); sabine.galindo@umontpellier.fr (S.S.-G.); caroline.strub@umontpellier.fr (C.S.); 2 CIRAD, UMR Qualisud, F-34398 Montpellier, France

**Keywords:** antagonistic agents, in vitro dual culture bioassay, mycotoxins, nutritional competition

## Abstract

The aim of this study was to investigate the impact of commercialized biological control agents (BCAs) against two major mycotoxigenic fungi in cereals, *Fusarium graminearum* and *Fusarium verticillioides*, which are trichothecene and fumonisin producers, respectively. With these objectives in mind, three commercial BCAs were selected with contrasting uses and microorganism types (*T. asperellum*, *S. griseoviridis*, *P. oligandrum*) and a culture medium was identified to develop an optimized dual culture bioassay method. Their comportment was examined in dual culture bioassay in vitro with both fusaria to determine growth and mycotoxin production kinetics. Antagonist activity and variable levels or patterns of mycotoxinogenesis inhibition were observed depending on the microorganism type of BCA or on the culture conditions (e.g., different nutritional sources), suggesting that contrasting biocontrol mechanisms are involved. *S. griseoviridis* leads to a growth inhibition zone where the pathogen mycelium structure is altered, suggesting the diffusion of antimicrobial compounds. In contrast, *T. asperellum* and *P. oligandrum* are able to grow faster than the pathogen. *T. asperellum* showed the capacity to degrade pathogenic mycelia, involving chitinolytic activities. In dual culture bioassay with *F. graminearum*, this BCA reduced the growth and mycotoxin concentration by 48% and 72%, respectively, and by 78% and 72% in dual culture bioassay against *F. verticillioides*. *P. oligandrum* progressed over the pathogen colony, suggesting a close type of interaction such as mycoparasitism, as confirmed by microscopic observation. In dual culture bioassay with *F. graminearum*, *P. oligandrum* reduced the growth and mycotoxin concentration by 79% and 93%, respectively. In the dual culture bioassay with *F. verticillioides*, *P. oligandrum* reduced the growth and mycotoxin concentration by 49% and 56%, respectively. In vitro dual culture bioassay with different culture media as well as the nutritional phenotyping of different microorganisms made it possible to explore the path of nutritional competition in order to explain part of the observed inhibition by BCAs.

## 1. Introduction

Many plant pathogens endanger agriculture, particularly in cereal production. Some of these phytopathogens can produce mycotoxins and have multi-destructive effects through reducing the yield and nutritional properties of grain, and can also affect its health-related characteristics. Among mycotoxigenic fungi, the genus *Fusarium* is the most prevalent and represents a significant risk [1].

Mycotoxins are small toxic molecules resulting from the secondary metabolism of these fungi. *Fusarium graminearum* is able to produce deoxynivalenol (DON) and its derivatives 15 and 3-ADON. *Fusarium verticillioides* mainly produces fumonisins B_1_ and fumonisins B_2_ (FB_1_ and FB_2_). They are ubiquitous in agricultural production, and cereal crops are the primary source of consumer exposure to mycotoxins [2]. Mycotoxins are quite stable molecules which are very difficult to remove or degrade, and are found along the food chain while retaining their toxic properties. They can cause acute and chronic intoxication in both humans and animals depending on different factors (intake levels, duration of exposure, toxin type, mechanisms of action, metabolism, and defense mechanisms) [3]. The symptoms can be very variable depending on organisms considered, but can range from food poisoning or gastric-intestinal problems to suppression of the immune system, the induction of cancer, or death [4,5]. Sometimes the combination within trichothecenes/fumonisins or with other mycotoxins such as ochratoxins or aflatoxins can produce a synergistic toxic effect [6,7]. Given their high toxicity, DON and fumonisins have been regulated by the European Union and the maximum recommended levels of mycotoxins are 750 µg kg^−1^ and 1000 µg kg^−1^, respectively, for processed cereal intended for human consumption (Commission Regulation (EC) No 401/2006). Economically, these contaminants hamper international trade and significantly affect the world economy. Because their appearance can occur before or after harvesting, one of best ways to reduce the presence of mycotoxins in food and feed is to prevent their formation in the crop [8]. 

Agricultural production is subject to a scissor effect with, on one hand, very evolutive climatic conditions that promote microbial development and, on the other hand, the political determination to reduce the use of phyto-chemical products. The adverse effects of plant protection products on environment and on human and animal health have encouraged the European Union to promote the search for alternative and environmentally friendly solutions such as integrated pest protection and the use of biological control agents (BCAs) [9]. Non-pathogenic, they can prevent or reduce the progression of these fungi through different mechanisms. These mechanisms are very diverse, direct or indirect, and can be used together or alone depending on the biocontrol agent. The main direct mechanisms may be related to antibiosis, competition, parasitism, or toxin biotransformation [10]. Antibiosis includes the production of secondary metabolites like antibiotics, cell wall-degrading enzymes, and molecules that can suppress growth or kill pathogens [11,12,13]. Competition can occur when two or more fungi are present simultaneously. Microorganisms can then enter into nutritional competition when they need the same essential nutrient, or into spatial competition when they occupy the same limited space [14,15]. Parasitism is a direct attack of pathogen by the BCA that will degrade or consume the pathogen host until it dies [16]. Some mechanisms can be indirect. Colonization of the plant by beneficial microorganisms can trigger local or systemic defensive responses, enhancing resistance against phytopathogens [17,18].

Although the production of mycotoxins by toxigenic pathogens is of economic importance, many studies do not take this into account when studying biological control strategies. These studies are then limited to the fungicidal or fungistatic effects of BCAs, while the effect of BCAs on mycotoxin production is often neglected [19]. Moreover, most studies on the biocontrol of other plant pathogens do not consider the potential presence of several mycotoxins simultaneously in the crop, nor the side effects of BCAs on the mycotoxigenic agents present. Despite more than 70 years of intensive research and some promising results, only a few BCAs are currently available on the market, and for *Fusarium spp*. for instance in Europe, only *Pseudomonas chlororaphis* and *Pythium oligandrum* are available, as the commercial products Cerall^®^ (Belchim Crop Protection) and Polyversum^®^ (Biopreparáty/De Sangosse), respectively.

The lack of commercially available BCAs for mycotoxigenic fusaria may be due to numerous factors (unstable biocontrol efficacy under field conditions, strict storage and transport conditions for BCAs, complexity of registration) and more importantly a lack of knowledge about biocontrol mechanisms [20], especially concerning mycotoxins.

Indeed, a better understanding of the mechanisms that allow the biocontrol of pathogens, especially on marketed agents that have passed all the stages of formulation and registration, will make it possible to identify particularly effective mechanisms or biomarkers of mechanisms. This information could be used to optimize the use of existing BCAs but also to refine the selection of new BCAs. 

The ultimate goal of the present study was to characterize the impact of commercial biocontrol agent on life traits of mycotoxigenic fungi (growth and mycotoxinogenesis). Therefore, the objectives were (1) to develop optimal methods of dual culture bioassay, allowing the analysis of the interaction between pathogens and BCAs; (2) to identify effect of BCAs on growth and mycotoxins of *Fusarium graminearum* and *Fusarium verticillioides*; and (3) to propose a hypothesis on the modes of action of BCAs against mycotoxigenic pathogens. With these objectives in mind, three commercial BCAs were selected with contrasting uses and microorganism types (*T. asperellum*, *S. griseoviridis*, *P. oligandrum*) and studied with in vitro dual culture bioassay against *F. graminearum* and *F. verticillioides* with an optimized method promoting mycotoxin production. Variable levels of mycotoxinogenesis and growth reduction were observed depending on the microorganism type of BCAs or on the culture conditions (e.g., different nutritional sources), suggesting contrasting biocontrol mechanisms. Macroscopic and microscopic observation of interactions support this hypothesis, finding various structural formations of BCA. Nutritional phenotyping was used to detect the possibility of nutritional competition between microorganisms, and to identify the trophic requirements of BCAs. 

## 2. Results

To permit the interaction between BCAs and pathogens during dual culture bioassay, the first step was to select appropriate media to allow the development of each microorganism’s pairing (BCA/pathogen). BCA/pathogen selection criteria and descriptions are presented in Section 5, Material and Methods. Then, to characterize this interaction on selected media, integrative approaches were used. Microscopic and macroscopic observations were undertaken to identify specific comportment of BCAs and their effects on *Fusarium* physiology. The BCA treatment’s impact on growth, global mycotoxins, and specific mycotoxin production kinetics of pathogens was analysed. Finally, the impact of differential nutritional resources during dual culture bioassay was analysed and linked with the nutrient profiling of all microorganisms.

### 2.1. Comportments of Microorganisms and Culture Media Selection

**Figure 1:**
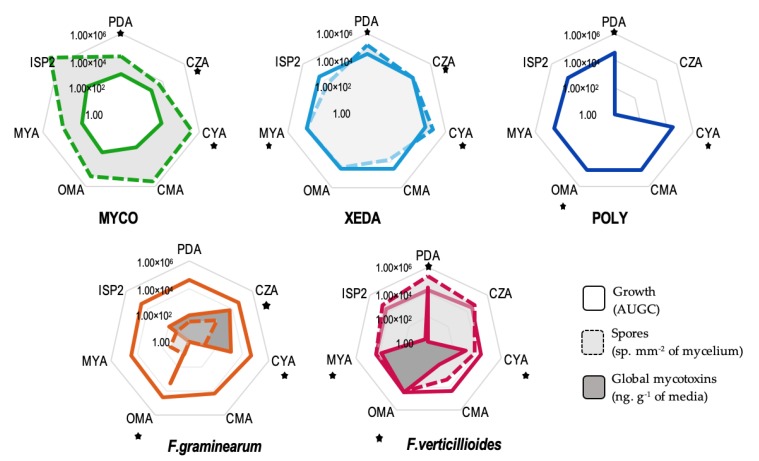
Comportment of all micro-organisms (commercialized biological control agents (BCAs) and pathogens) on different media. Stars indicate the final selected medium for each microorganism. Myco: Mycostop^®^, Xeda: Xedavir^®^, Poly: Polyversum^®^. AUGC: area under the growth curve, PDA: potato dextrose agar, CZA: Czapeck agar, CYA: Czapeck yeast agar, CMA: corn meal agar, OMA: oat meal agar, MYA: malt yeast agar, ISP2: International *Streptomyces* Project Medium 2 (8 days at 25 °C). Levels of global mycotoxins are given as a sum of the mycotoxins from a pathogen chemotype (*Fusarium graminearum*: deoxynivalenol (DON) + 15ADON and *Fusarium verticillioides*: fumonisins B_1_ (FB_1_) + fumonisins B_2_ (FB_2_)).

After 8 days of incubation, all microorganisms were able to grow on all different media, with the exception of Polyversum^®^ (Poly), which was unable to grow on Czapeck agar (CZA) synthetic medium (Figure 1). Mycostop^®^ (Myco), which has the most limited growth (area under the growth curve, AUGC < 1.85 × 10^2^), was an excellent spore producer (spores > 3.85 × 10^3^), especially on International *Streptomyces* Project Medium 2 (ISP2). Xedavir^®^ (Xeda) was minimally affected by the culture medium variation. Polyversum^®^ (Poly), the oomycete BCA, was characterized solely on growth capacity because oospore obtention was a long and complex process which is difficult to achieve, and to obtain oospores under these specific in vitro conditions. *F. graminearum* produced a low quantity of spores affected by medium variation. For both pathogens, global mycotoxin production was a very discriminant variable for medium selection. The culture media on which pathogens *F. graminearum* and *F. verticillioides* produced the most mycotoxins were respectively CZA and PDA. 

In order to promote pathogen development and mycotoxin production during interaction, the selection of dual culture bioassay media was firstly based on the mycotoxin production capacity of pathogen (Table 1). Then, the ability of BCAs for growth on this medium was checked. To observe the differential capacity of BCAs to impact mycotoxigenic fungi, one common medium (CM) for each pathogen was selected: for *F. graminearum*–BCA dual culture bioassay CYA was chosen, and for *F. verticillioides*–BCA dual culture bioassay PDA was selected. Other interesting media which did not allow all BCAs to grow were assigned to a specific dual culture bioassay duo (SM), and were selected for their ability to promote mycotoxinogenesis. These selected culture media were used to test antagonist activity and allowed identification of the impact of BCAs on pathogenic growth and mycotoxin production. The use of different culture media enabled the characterization of the impact of nutrient resources on the dual culture bioassay of microorganisms.

### 2.2. Macroscopic and Microscopic Interaction

**Figure 2:**
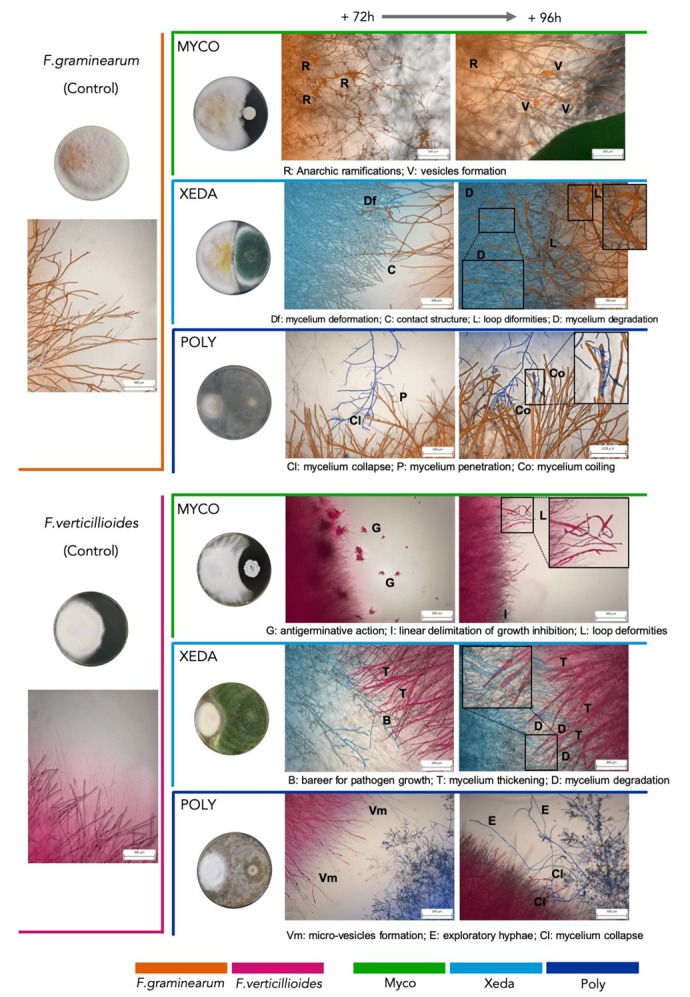
Macroscopic and microscopic interactions during pathogen–BCA dual culture bioassay. The *F. graminearum* and *F. verticillioides* control and confrontation plates respectively had CYA and PDA media (CM, 7 days after inoculation). The same media were used for the slide confrontation (+72/96 h after inoculation) indicated at the top of the figure. Colors indicate the type of microorganisms, and letters indicate specific structures. Myco: Mycostop^®^, Xeda: Xedavir^®^, Poly: Polyversum^®^.

During the dual culture bioassay test, the macroscopic observation of plates (7 days after inoculation) revealed differential comportments between microorganisms (Figure 2). For both pathogens, mycelial development was limited by the presence of all BCAs compared to the control. Myco formed a very small colony related to pathogens, but an inhibition zone of pathogen growth was observed (especially in dual culture bioassay with *F. verticillioides*). With respect to pathogens, accumulation of pigment was observed in the dual culture bioassay zone (on the reverse side of Petri dish, not shown). The Xeda colony grew as fast as the pathogen and filled the free space in the Petri dish. Once again, accumulation of pathogenic pigment in the confrontation zone could be observed on the reverse side of Petri dishes. The Xeda colony could exude fine black droplets on its surface solely during confrontation. Poly was able to grow faster than the pathogen (especially faster than *F. graminearum*) and fill the free space in Petri dish. During co-culture, it was able to progress on both the pathogenic colony and inhibit the homogenous and natural pink pigmentation of *F*. *graminearum* completely. 

At the microscopic scale, *F. graminearum* alone produced rapidly long, dense, and straight hyphae, while *F. verticillioides* alone produced fine and extra fine lightly branched hyphae (Figure 2). With microscopic interactions between BCAs and pathogens photographed over time, many characteristic structures could be observed. Myco was able to cause anarchic ramifications of *F. graminearum* mycelia before contact (+72 h) and vesicle formation at the tip of pathogen hyphae when they were close (+96 h). Growth inhibition by Myco was clearly identified at the microscopic level in co-culture with *F. verticillioides*, with an early antigerminative action (+72 h) and a linear/regular growth inhibition after 96 h. Xeda induced deformation of *F. graminearum* mycelia, which could extend to the formation of loops after 96 h. The progression of this BCA face-to-face with *F. verticillioides* created a mechanical barrier that slowed the progression of the mycotoxigenic fungus. In the dual culture bioassays of both pathogens, Xeda caused a degradation of pathogenic mycelia in contact zones (+96 h). The co-culture *F. graminearum*–Polyversum showed dispersed contact structures. The BCA was able to pass through the mycelium of the pathogen, and induced formation of lysed wall hyphae. It adopted a completely different comportment in the dual culture bioassay against *F. verticillioides* and induced the transient formation of micro-vesicles at the extremity of pathogenic hyphae (between 24 and 72 h of dual culture bioassay). When it was close to the pathogen (+96 h), it deployed long mycelial filaments to colonize the environment.

### 2.3. BCA Impact on Growth and Mycotoxin Production

**Figure 3:**
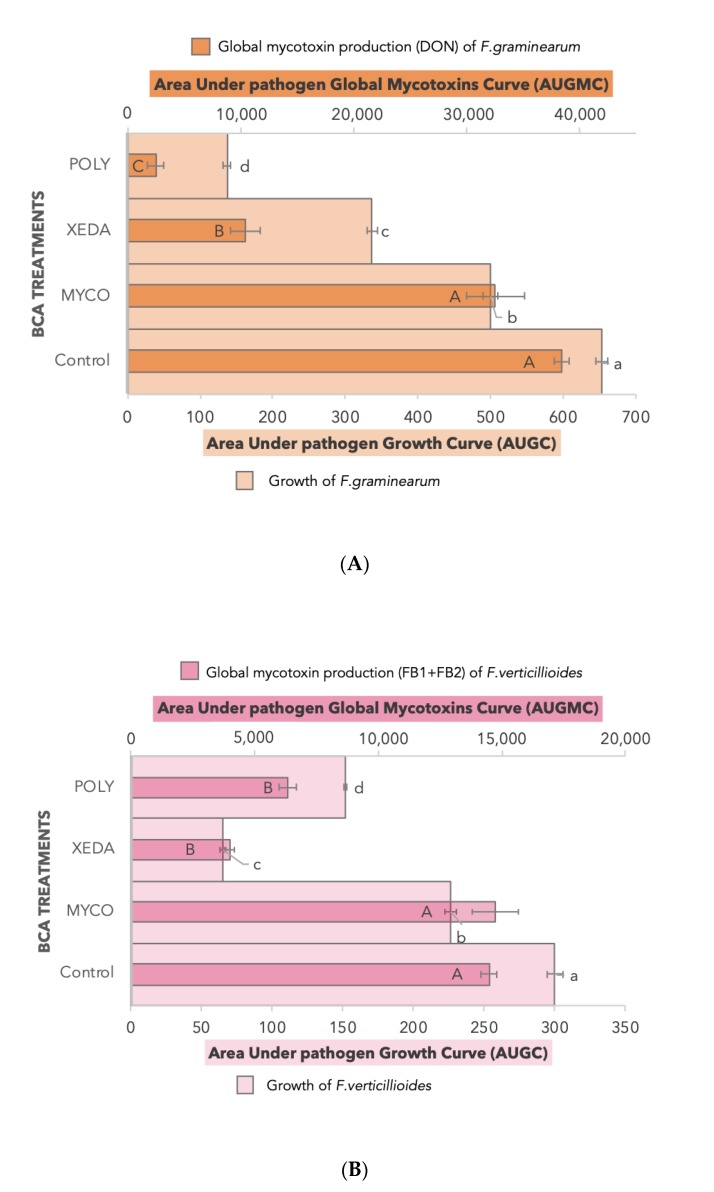
Comparative effects of BCA treatments on the evolution of pathogens. Dual culture bioassays for all BCAs against one pathogen were performed on common medium (CM). (**A**) *F. graminearum*, on CYA, in orange; (**B**) *F. verticillioides*, on PDA, in pink, for 12 days. Myco: Mycostop^®^, Xeda: Xedavir^®^, Poly: Polyversum^®^. Growth (pastel colors, bottom axis) and mycotoxin production levels (pastel colors, top axis) are expressed in the area under growth/global mycotoxin curves (respectively AUGC and AUGMC). Original kinetic curves are available in Figure A2. ANOVA test, Growth comparisons (a, b, c, d) and Mycotoxin comparisons (A, B, C). *p*-value < 0.05. FB_1_ + FB_2_ are represented together because the quantity of FB2 was not significant.

During the antagonistic in vitro test, the growth and the mycotoxin production of both pathogens in confrontation against the three BCAs were monitored over 12 days. Classic batch curves were obtained for pathogen growth and major mycotoxin production (latent phase, exponential phase, stationary phase). The area under growth/global mycotoxins curve (respectively AUGC and AURMC) was calculated (Figure 3). All BCA treatments impacted growth of *F. graminearum*, especially Xeda and Poly, causing growth reductions of respectively 48% and 79%. 

In addition to reducing the pathogens mycelial development, Xeda and Poly had a direct action on global DON production, with reductions in mycotoxin concentrations of 73% and 93% respectively. *F. graminearum* 15-ADON production was affected and complete inhibition of 15-ADON peak production (8 days after inoculation) was observed with all BCA treatments (see Figure A2). Dual culture bioassays with *F*. *verticillioides* showed a completely different response profile to BCAs. Even if the growth reduction of pathogen was significant face-to-face with Myco (24%), the specific production was lightly stimulated. It was mainly with the reduction of pathogen growth that Xeda restricted fumonisin production, with the most important inhibition levels (78%) among all BCA tested. 

In summary, Poly was more efficient in the *F*. *graminearum* dual culture bioassay and Xeda was more efficient in the *F*. *verticillioides* dual culture bioassay using common media (CM).

### 2.4. Impact of Media Variation on Pathogenic Factors during Dual Culture Bioassay (Pathogens–BCAs)

**Figure 4:**
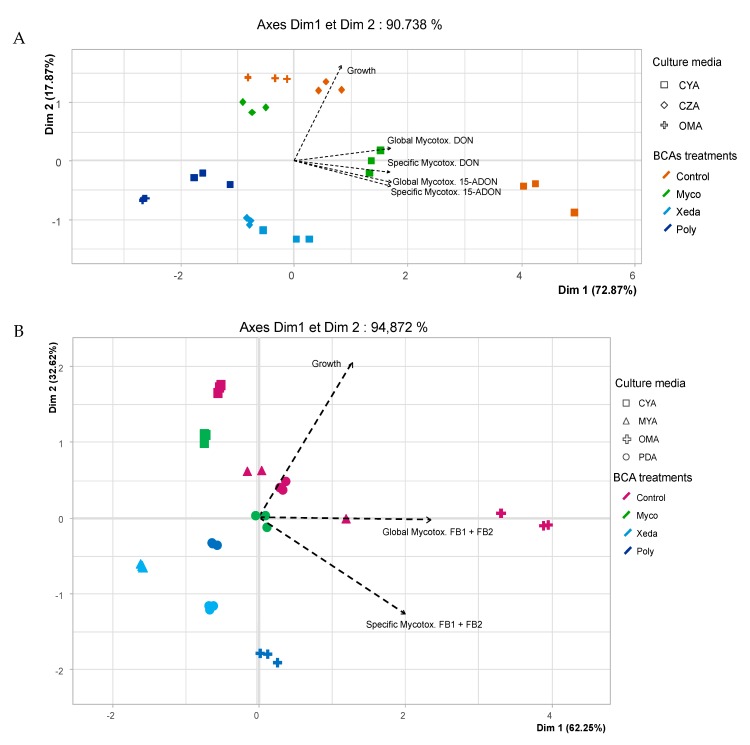
Principal component analysis (PCA) bi-plot with respect to the growth and mycotoxin production data set (---) for the two considered pathogens, (**A**) *F. graminearum* and (**B**) *F. verticillioides*, obtained after BCA confrontation (indicated by colors) in different selected culture media (indicated by forms). Myco: Mycostop^®^, Xeda: Xedavir^®^, Poly: Polyversum^®^.

Principal component analysis (PCA) analysis was performed in order to visualize global inhibition profiles of pathogens depending on BCA treatment, but also to assess the impact of culture medium on those inhibition profiles (Figure 4). For both biplots, the variables are well-projected and equivalently projected, and the two first components explained 90.7% and 94.8% of data distribution for *F. graminearum* (Figure 4A) and *F. verticillioides* (Figure 4B), respectively. The growth factor mainly contributed to Dim2, mycotoxin factors mainly contributed to Dim1, and all variables were in correlation. With all culture media combined, Myco and Control samples were regrouped in both graphs in the positive part of biplot. However, culture media influenced the separation of samples on CZA (*F. graminearum* control or confronted with Myco). Control samples were correlated with DON and 15-ADON factors, in contrast to Myco treatment samples, on the other side of Dim2. In the *F. graminearum* bi-plot (Figure 4A), Xeda and Poly samples are regrouped in the bottom-left quarter of graph, suggesting strong opposition to the studied pathogen factor. For the Poly-confronted samples, an increase of mycotoxin inhibition was observed in OMA, highlighting the important effect of culture media on dual culture bioassay, illustrated here for *F. graminearum*. Global antagonist activity of Xeda against *F. graminearum* in the CYA medium was higher than on CZA. On the *F. verticillioides* bi-plot (Figure 4B), these two BCAs (Xeda and Poly) caused the most antagonistic activity. However, against this pathogen, Xeda showed the strongest growth and mycotoxin inhibition. MYA-confronted samples were further from the specific mycotoxin production factor than PDA-confronted samples, indicating a higher specific mycotoxin reduction in this culture medium. Poly was able to inhibit pathogenic factors in both culture media, but with OMA the separation between control and confronted samples was greater, suggesting a stimulation of antagonistic capacities on this culture medium.

### 2.5. Nutrient Profiling and Nutritional Competition

**Figure 5:**
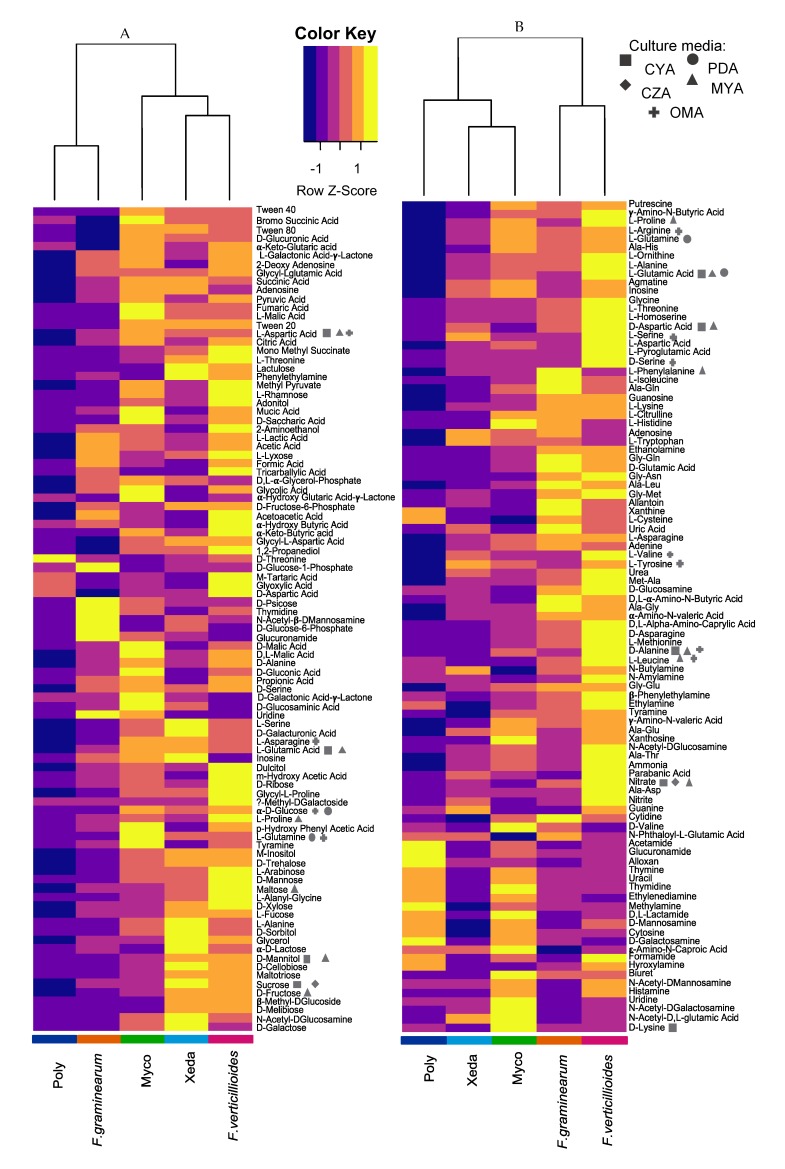
Growth induction of microorganisms during growth on 95 carbon sources (PM1) (**A**) or 95 nitrogen sources (PM3) (**B**). Both panels show optical density measurements over a 138-h time course (i.e., area under the growth curves, AUGCs). Color keys of the heat map (from purple to yellow) are expressed for one nutrient in comparison with other microorganisms (row comparison). Micro-organisms are indicated by colors at the end of each column. Myco: Mycostop^®^, Xeda: Xedavir^®^, Poly: Polyversum^®^. Major elements included in the composition of selected media are indicated by forms next to the concerned nutrient. Medium compositions are available in Figure A1.

Given the observed medium impact, nutrient profiling of all microorganisms was characterized using phenotype microarray, and a comparison between microorganisms was performed to identify potential nutritional competition. Along 138 h of incubation, a contrasting capacity to consume the different nutritional resources appeared. As shown in Figure 5, globally, two clusters of microorganisms can be distinguished based on the capacity to metabolize C sources (Figure 5A), with separate microorganisms in two clusters: one with Poly and *F. graminearum*, which were able to consume less C sources than others (high trophic requirements), and one including *F. verticillioides*, Xeda, and Myco, which could consume a broad range of C sources (low trophic requirements). Some C compounds strongly stimulated the growth of microorganisms, such as tyramine/glucose, N-acetylglucosamine/sucrose, threonine/tartaric acid, glycerol/uridine, and proline/sucrose, respectively, for Myco, Xeda, Poly, *F. graminearum*, and *F. verticillioides*. Sucrose was therefore the carbohydrate allowing the strongest growth of two antagonistic microorganisms (Xeda and *F. verticillioides*). On other hand, threonine is a compound that is consumed only by the Poly BCA and inhibits the growth of other microorganisms, including the two mycotoxigenic pathogens. Concerning the aptitude of microorganisms to degrade nitrogen sources (Figure 5B), two different clusters were formed: one with of Poly, Xeda, and Myco, which were able to consume less N sources than others (specialized); and one including *F. graminearum* and *F. verticillioides*, which could consume a broad range of N sources (unspecialized). This clustering was in accordance with the type of microorganism (BCAs or pathogens), and Poly has a nitrogen consumption profile which is in complete contrast to the *F. verticillioides* profile. Some N nutrients increase the growth of particular microorganisms: histidine/lysine, uric acid/serine, xanthine/L-cysteine, uric acid/allantoin, and serine /L-pyroglutamic acid, respectively, for Myco, Xeda, Poly, *F. graminearum*, and *F. verticillioides*. Preferential nitrogen sources of Xeda were also the preferential nitrogen sources of the two pathogens. 

Nutrient consumption and the resulting growth capacity were difficult to relate to the synergistic effects that could be observed in the dual culture bioassay on complex synthetic media. Nevertheless, results show that Poly, because of its high trophic requirement, has more difficulty consuming nutrients than pathogens. This observation is confirmed with regard to the major elements that comprised the culture media of the dual culture bioassay (the two CM pathogens PDA and CYA, and the SM BCAs OMA), even on OMA, where it showed very strong antagonistic capacities during dual culture bioassay. Xeda had better biocontrol activity against *F. verticillioides* on MYA (global mycotoxin inhibition), and against *F. graminearum* on CYA (growth and global mycotoxin inhibition). The MYA medium was composed of fructose and mannitol, which the pathogen/BCA duo can degrade in equivalent proportions. CYA medium contains sucrose and nitrate, two compounds for which Xeda has a stronger affinity than *F. graminearum.*

## 3. Discussion

Based on the presented results, the development of dual culture bioassay methods and in particular the choice of culture media should be considered during in vitro studies between mycotoxigenic pathogens and BCAs. Globally, complex culture media selected for the affinity that the microorganisms have for them allow good growth of all microorganisms, with the exception of Poly which does not grow on CZA, a minimum medium. However, the mycotoxinogenesis of pathogens is strongly influenced by the type of media, as shown by our results and as widely reported in the literature [21,22]. A study conducted by Gardiner et al. [22] on the nutritional profile of *F. graminearum* identified the same type of profile, with preferential consumption of the same nutrient as in our results. It is therefore necessary to select culture media that will promote production of this mycotoxin in order to identify BCAs that will have a considerable impact even during high production. Most of the in vitro dual culture bioassays between pathogenic fungi and BCAs are performed on PDA [23,24], which does not stimulate the production of tricothecenes by *F. graminearum*. The use of two media per BCA/pathogen coupling allows the observation of whether nutritional conditions impact the inhibition caused by BCAs. The high mycotoxigenic capacity of the strains was verified. Many variations can be observed between the two pathogens. In natural conditions, they originate from different environments, as *F. graminearum* is isolated from wheat spikes while *F. verticillioides* is isolated from maize kernels. In addition, the pathogens produce distinct types of mycotoxins (trichothecene for *F. graminearum* and fumonisins for *F. verticillioides*) and their production levels are also different. According to the analyses that we performed on both pathogenic strains, the maximum production of fumonisin and trichothecene was about 5000 and 15,000 ng g^−1^ of media, respectively. Moreover, their colonization strategies are in contrast: *F. graminearum* grew rapidly while producing fewer spores, unlike *F. verticillioides* for which expansion was slower, but it compensated with a substantial production of spores, allowing it to colonize the environment in a comparable way. For these reasons, most of the analyses are carried out independently.

In dual culture bioassay with pathogens, Myco did not have time to develop, which did not allow it to compete spatially with pathogens. The halos of inhibition and the absence of contact during the observation period as well as the deformation of the pathogenic hyphae of *F. graminearum* suggest the synthesis of diffusible antimicrobial compounds by the BCA. Actinomycetes, and particularly *Streptomyces* species, are well known for their production of a wide spectrum of antibiotics [25,26]. In dual culture bioassay with *F. verticillioides*, they were even able to remotely prevent germination of pathogenic spores and completely inhibit pathogen progression. Several compounds were emitted by *Streptomyces spp*. and identified (e.g., as methyl vinyl ketone) for their ability to inhibit the spore germination of phytopathogens [27,28]. The appearance of vesicles at the tips of the hyphae of *F. graminearum* may be related to the synthesis of mycotoxins. Some studies have found that mycotoxins, notably in *F. graminearum*, were synthesized in toxisomes, a kind of vesicle excreted out of the cells and at the tip of hyphae [29,30]. The pathogen’s perception of BCA may stimulate this production. Based on the results of the antagonistic tests, Myco tends to stimulate specific mycotoxin production despite significant inhibition of the growth of both pathogens. Therefore, it is essential to test the ability of BCAs to impact mycotoxin production and not only growth. Its ability to degrade many sources of C allows it to grow under many conditions, which could make it a preventive treatment. However, to effectively control pathogens, the ratio of inoculum from Myco must be superior than the quantity of pathogens. 

Xedavir adopts a completely different strategy. With good colonization capacities, can compete with the development of pathogens. It can form a mechanical barrier that physically blocks the development of pathogenic fungi and is also be able to establish connections between its hyphae and those of pathogens through the formation of haustaurium [31]. When in contact with pathogens, it can also synthesize compounds that degrade the fungal walls, probably chitinases, preventing the progression of pathogens. Numerous studies have shown this ability in different species of *Trichoderma* [32,33]. Not surprisingly, the genomes of the mycoparasitic *Trichoderma spp*. are rich in gene-encoding enzymes like chitinases and glucanases [34,35]. This wall degradation will provide a preferential substrate for further development. It will rather have a hyperparasitic behavior [36]. Its goal is to kill pathogens. Faced with *F. graminearum*, it will act directly on the specific mycotoxinogenesis independently of growth reduction. It will therefore be able to act directly on the mycotoxin biosynthesis pathways, or by biotransformation of the mycotoxins produced (transformation, degradation, binding). It would be interesting to explore this potential because recent studies have identified mycotoxin degradation capacities in other BCAs of the *Trichoderma* genus [37]. During in vitro dual culture bioassays on selected media, culture media seem to influence the impact of Xeda on pathogens. The nutritional resources of microorganisms will therefore impact the inhibition of pathogens by BCAs. One culture medium could even allow Xeda to act more drastically on specific mycotoxinogenesis (MYA–*F. verticillioides*), and thus reduced the mycotoxin concentrations of samples. These results suggest that nutritional competition phenomena could be one of the weapons used by Xeda to fight pathogens. This path is supported by the comparison of nutritional profiles of Xeda and pathogen nutritional profiles. It appears that for certain compounds, the capacities of assimilation will be equivalent or to the advantage of BCAs, notably certain culture media compounds used during the dual culture bioassays (like sucrose). Indeed, culture media that allow a better inhibition of pathogens (MYA and CYA, respectively for *F. verticillioides* and *F. graminearum*) will be considered as resources for which microorganisms are in competition. Recently, Wei and collaborators showed that root-associated bacterial communities with a clear niche overlap with the pathogen reduce pathogen invasion success, constrain pathogen growth, and have lower levels of diseased plants in greenhouse experiments [38].

Concerning interactions between Poly and fusaria pathogens, Poly is one of the most competitive BCAs despite its high nutritional requirements. It is able to proliferate very rapidly under favorable conditions. Against the two pathogens, it will be able to invade the space left free more quickly, until it grows on the colonies of the pathogen. This behavior suggests an interaction requiring close contact, like parasitism. Poly is known for its capacities to colonize fungi and oomycetes [39]. The perception of the pathogen could be done via the perception of ergosterol from pathogens. In fact, sterols have been identified first as stimulating the sexual reproduction of *Pythium* species (like Poly) [40,41,42]. The same stimulation effects have been observed during dual culture bioassays with fungal pathogens [43]. This ability is a considerable advantage for the invasion of Poly and its mechanisms of action against pathogens. In addition to limiting the spatial spread of the pathogen, the absence of pathogenic pigment suggests a direct interaction with the metabolism of mycotoxigenic pathogens. At the same time after inoculation, compared to Xedavir, which has a slower progression, the contact took place in a very subtle manner. It seems that the BCA did not want to be perceived by the pathogens and only few wall degradation enzymes were synthesized. In dual culture bioassay against *F. graminarum*, Poly was able to pass through the mycelium of the pathogen and induce formation of lysed wall hyphae. They could be considered as privileged spaces for nutrient sampling by the BCA, suggesting again a close-contact interaction, such as mycoparasitism. These observations are entirely consistent with another study by Charlène Faure et al., who had observed the same type of behavior by Poly, capable of crossing the mycelium of *F. graminearum* without forming any particular structure [43]. During the antagonist activity test, Poly showed very good antagonistic capacities, allowing a reduction of the growth of both pathogens. However, the impact on mycotoxin production was much stronger on *F. graminearum*, against which it was particularly effective. By integrating the impact of the growing medium, Poly was the BCA that was most sensitive to change in nutritional resources. From the beginning of the study, it was the microorganism that was the most sensitive to culture media, with an inability to produce oospores in vitro in synthetic culture media that are not enriched in sterols. In contrast, it was able to deploy its mechanisms of action during dual culture bioassays because it was stimulated by the presence of the pathogen and by the complex nutrients in the culture media. In fact, it was most effective on a culture medium composed mainly of cereals (OMA). The results showed that Poly was less adapted in terms of nutrient consumption than the pathogens on all the culture media of the dual culture bioassays, even on OMA, where it showed very strong antagonistic capacities during dual culture bioassay test. It would therefore appear that unlike Xeda, nutrient competition was not a preferential mechanism for Poly to deal with mycotoxigenic fungal pathogens. Nevertheless, it is interesting to note that threonine was a compound that was consumed only by Poly BCA and did not promote growth of other microorganisms, including the two mycotoxigenic pathogens. It was interesting to test the enrichment of commercial formulations with compounds of this type in order to boost the growth of Poly during treatment in real conditions. 

The three BCAs therefore have contrasting and complementary strategies, with different capacities to inhibit the propagation of pathogens. Their behavior was identical in one aspect: all inhibited the peak production of 15-ADON produced by *F. graminearum* (see Figure A2A). 15-ADON is a precursor to the formation of DON [44]. This inhibition could therefore be a sign of an action on the biosynthesis pathway, in particular the *Tri8* gene involved in the transformation of 15-ADON into DON. 

## 4. Conclusions

In the present study, three commercial biological control agents (BCAs) were selected and tested to evaluate their antagonistic activities against *F. graminearum* and *F. verticillioides*, two mycotoxigenic fungi. Results suggest that this three BCAs revealed contrasting effects on growth and mycotoxin production of pathogens, particularly Xedavir^®^ against *F. verticillioides* and Polyversum^®^ against *F. graminearum*, the two most effective combinations. In addition, several paths could be explored concerning the various observed modes of action of these BCAs, like antibiosis, parasitism, or mycotoxin degradation. It would appear that the inhibition effect of BCAs could be dependent on the nutritional condition of interaction, providing new insight into the nutritional phenotype and potential competition between micro-organisms. Given the high levels of inhibition observed, further studies on the modes of action of these BCAs could be conducted, for example on synthesis of anti-germinative compounds by Mycostop^®^, chitinolytic activities or by-products of microbial detoxification of mycotoxins by Xedavir^®^, or mycophageous action by Polyversum^®^.

## 5. Material and Methods

### 5.1. Micro-Organisms

#### 5.1.1. *Fusarium* Species

*F. graminearum* isolate BRFM 1967 and *F. verticillioides* isolate BRFM 2251 were used (CIRM, University of Aix-Marseille, Marseille, France), and chosen for their strong ability to produce their respective mycotoxins. *F. graminearum* strain BRFM 1967, isolated from wheat plant, has a deoxynivalenol (DON/15-ADON) chemotype profile, while *F. verticillioides* BRFM 2251, isolated from maize kernels, has a fumonisins (FB_1_/FB_2_/FB_3_) chemotype profile. All fungi were maintained on potato dextrose agar (PDA; BD Difco, Sparks, MA, USA) under paraffin oil at 4 °C and actively grown on PDA at 25 °C for 7 days for spore production.

#### 5.1.2. Commercial Biological Control Agents (BCAs)

Three commercial biological control agents were selected for their contrasting characteristics. To date, there is little information on their action and biocontrol mechanisms on mycotoxigenic fungi (Table 2).

All strains were isolated from their commercial product with a classical microbial insulation protocol (serial dilution and multiple striation inoculation), on appropriate medium (supplemented with 0.01% of tetracycline for Polyversum only). BCAs were conserved under spore forms in glycerol solution (15%/−80 °C) and in commercial product aliquots (4 °C). The strains were actively grown on ISP4, PDA, and V8, respectively, for Mycostop, Xedavir, and Polyversum at 25 °C for 7 days for spore production. For the rest of the study, the strains isolated from commercial products were referred to using the following abbreviations: Myco for Mycostop/*S. griseoviridis*, Xeda for Xedavir/*T. asperellum*, and Poly for Polyversum/*P. oligandrum*. 

#### 5.1.3. Behaviors on Different Media

**Figure A1:**
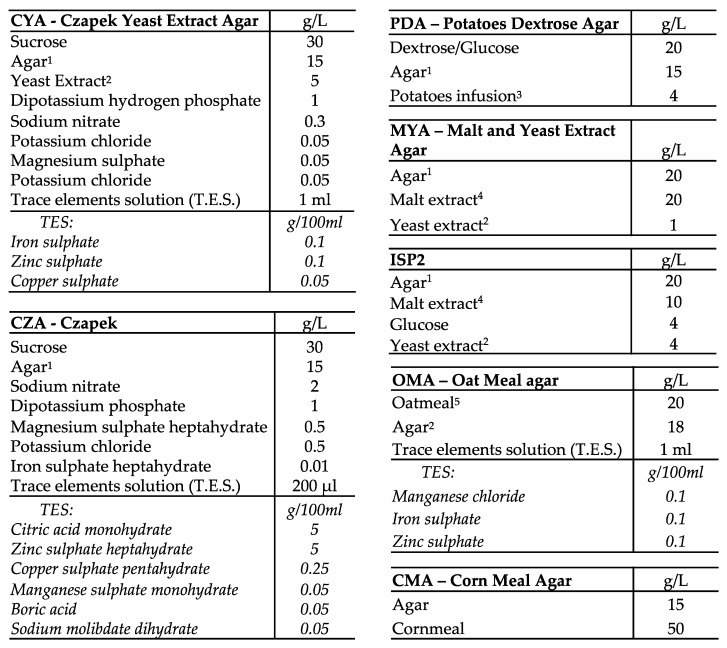
Detailed composition of culture media. In all tables, principal ingredients for the preparation of media in laboratory are described. Trace element solutions (TES) were added after autoclaving the culture media. For complex ingredients, superscript numbers refer to the main components in C and N identified through the literature. ^1^ Agar [59,60]. Major C components: β-D- galactopyrose, α-L-galactopyranose, agarobiose, agaropectine. Major N component: uronic acid. ^2^ Yeast extract [60,61]. Major C component: mannitol. Major N components: alanine, lysine, aspartic acid, glutamic acid. ^3^ Potato infusion [62,63]. Major C component: starch (glucose + glucose), Major N components: asparagine, glutamine, aspartic acid. ^4^ Malt extract [60,64]. Major C components: maltose, glucose-fructose, dextrine, maltobiose. Major N components: glutamic acid, proline, alanine, leucine, phenylalanine. ^5^ Oatmeal [65,66,67]. Major C components: starch (glucose+glucose), arabinoxylane, β-glucan. Major N components: phenylalanine, tyrosine, leucine, valine, arginine, lysine, glutamic acid, glycine, alanine, serine.

**Figure A2:**
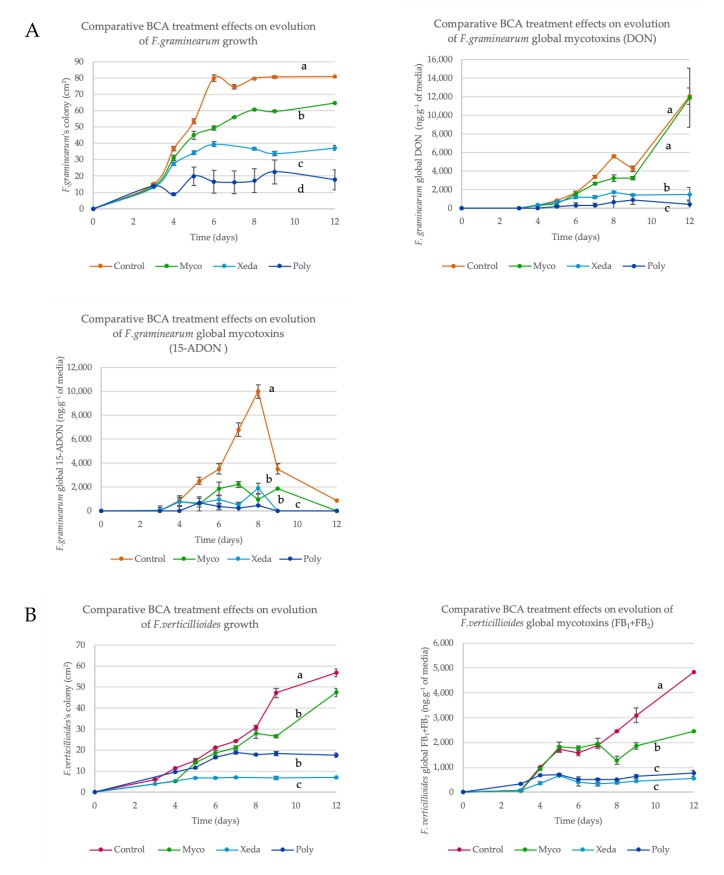
Comparative effects of BCA treatments on the evolution of pathogens. Dual culture bioassays for all BCAs against one pathogen were performed using common medium, CM. (**A**) *F. graminearum*, on CYA, in orange; (**B**) *F. verticillioides*, on PDA, in pink, for 12 days. Growth (A1, B1) is expressed in cm^2^, and mycotoxin production levels (A2, A3, B2) are expressed in ng g^−1^ of media. FB_1_ + FB_2_ are represented together because the quantity of FB2 was not significant. ANOVA test, *p*-value < 0.05.

To identify the culture media best suited to the dual culture bioassay of each of the BCAs/pathogen, seven culture media candidates were chosen: PDA, Czapeck agar (CZA), Czapeck yeast agar (CYA), and oat meal agar (OMA) for their affinity with pathogens [45,46,47,48], and potato dextrose agar (PDA), International *Streptomyces* Project Medium 2 (ISP2), malt yeast agar (MYA), and corn meal agar (CMA) for their affinity with BCAs (respectively all BCAs, Myco [49,50], Xeda [13,51,52], and Poly [53,54,55]). The compositions of media are available in Figure A1. All isolates were inoculated at the center of Petri dishes (5 μL × 10^4^ spores mL^−1^) and the growth kinetics were measured by image analysis on ImageJ software (area measurement in cm^2^/every two days for 8 days). Produced spores were evaluated by flooding the plates on the final day at the end of kinetics (after 8 days) and counted under an optic microscope with Thoma cell. Mycotoxin analyses were performed for pathogen with the method described below (Section 5.2.2).

This result was be used to select two culture media for each BCA/pathogen pair for the dual culture bioassay tests. 

### 5.2. Antagonist Activity in vitro on Selected Culture Media

#### 5.2.1. Dual Culture Bioassay and Growth Evaluation

On selected media (Table 1), one BCA and one pathogen were inoculated in a Petri dish, 85 mm in diameter, at the same distance from the center of plate. The pathogens and BCAs were inoculated with the spore suspension except for Poly, which was inoculated with 7-day plugs (5 mm in diameter). Each BCA was inoculated 24 h in advance (5 μL × 10^5^ spores mL^−1^ or plug); then the pathogens were inoculated at the opposite side (45 mm from BCA, 5 μL × 10^4^ spores mL^−1^). Plates were incubated at 25 ± 2 °C, in the dark, for 12 days. Plates inoculated only with the pathogen were used as a control treatment. Every two days, Petri dish were photographed, and the colony area of each BCA/pathogen were measured in cm^2^ by image analysis using ImageJ software (1.52a, Wayne Rasband National Institute of Health, Bethesda, MD, USA, 2018). Values were used to create growth curves. Each BCA/pathogen dual culture bioassay and control was set up in triplicate for each analyzed day and two independent repetitions of the test were done.

#### 5.2.2. Mycotoxin Extraction and Analysis

After growth evaluation, each dual culture bioassay plate was submitted to a mycotoxin analysis. The extraction procedure described by Moreau and Levi [56] was used with some modifications (no purification steps). For both pathogens, half a Petri dish was sampled by cutting along the line formed by the two inoculation points, and finely cut and weighed. For *F. graminearum* tests or controls, 50 mL of acetonitrile/water/acetic acid (79:20:1, *v*/*v*/*v*) were added. For *F. verticillioides* tests or controls, 150 mL of water/acetic acid (99.5:0.5, *v*/*v*/*v*) were added. All samples were homogenized by mechanical agitation for 20 min. *F. graminearum* samples were preliminarily diluted 1:50 in water/acetic acid (99.5:0.5; mobile phase of analyzer) and filtered with a CA filter (0.45 μm, Carl Roth GmbH, Karlsruhe, Germany); *F. verticillioides* samples were directly filtered, and were ready for injection.

For all samples, mycotoxin detection and quantification were achieved using an Ultra High-Performance Liquid Chromatography (UHPLC, Shimadzu, Tokyo, Japan) coupled with a mass spectrometer (8040, Shimadzu, Tokyo, Japan). LC separation was performed using a Phenemenex Kinetex XB Column C18 (50 mm × 2 mm; 2.6 μm particles) at 50 °C, with an injection volume of 50 μL. Mobile phase composition was (A) 0.5% acetic acid in ultra-pure water and (B) 0.5% acetic acid in isopropanol (HPLC MS grade, Sigma, St Louis, MO, USA), and the mobile phase flow rate was 0.4 mL min^−1^. The mass spectrometer was operated in electrospray positive (ESI+) and negative (ESI–) ionization mode, and two multiple reaction monitoring (MRM) transitions for each analyte were monitored for quantification (Q) and qualification (q) (Table 3, Table 4). All data were analyzed using LabSolution Software (v5.91/2017, Shimadzu, Tokyo, Japan,2017). Limits of detection or quantification (LOD/LOQ in ng mL^−1^, respectively) for each mycotoxin were: DON (4/14), 15-ADON (10/35), FB_1_ (0.03/0.1), and FB_2_ (0.01/0.05). Mycotoxin levels were expressed in ng g^−1^ of medium or ng cm^−2^, respectively, for global and specific mycotoxin production. Values were used to create global and specific mycotoxin production curves. Each BCA/pathogen dual culture bioassay and control mycotoxin analysis was set up in triplicate for each measurement day, and two independent repetitions of the test were done.

### 5.3. Macroscopic and Microscopic Observations

For the macroscopic observations, plates obtained in the same conditions described previously were used to identify for signs of an inhibition zone between each BCA/pathogen dual culture bioassay or/and the activation of secondary metabolism through the differential presence of pigments compared to the control or/and the BCA overgrowth on the pathogenic colony suggesting mycoparasite activity. The BCA control plates were added, with BCA alone in same conditions.

Microscopic observations were performed with an adapted method from Reithner [57]. On glass slides, 1.5 mL of common medium (CM) for each BCA/pathogens pair (CYA for *F. graminearum* and PDA for *F. verticillioides*) were deposited, inoculated with BCA/pathogen pairs on opposite sides (4.5 cm), incubated at 25 °C on supports in Petri dishes containing wet sterile filter paper, and sealed with parafilm. Glass slides inoculated only with pathogens were used as the control. The evolution of interaction was observed daily until 24 h after contact between BCAs and pathogens (120 h), with a Zeiss PrimoStar microscope. Pictures were taken using a Zeiss Axiocam ERc5s camera (Carl Zeiss Microscopy, Thornwood, NY, USA). The obtained pictures were treated with Photoshop CC Software (19.0, Adobe, San Jose, CA, USA, 2017), using the following pipe-line: contrast/light correction, filter layer correction, selection of a color/black and white density filter (reduces yellowing of color treatment, provides contrast for the microorganism contours), complementary manual selection of microorganisms, color filling (background transparency and Gaussian blur), and directional gradient in section.

### 5.4. Nutrient Profiling

Phenotype microarrays plates (Biolog, Hayward, CA, USA) were used for the nutrient profiling of all microorganisms according to the manufacturer’s instructions. For the carbon and nitrogen consumption profiling, PM1 and PM3 were used, respectively. Carbon sources in PM1 are in the 5–50 mM range and nitrogen sources in PM3 are in the 2–20 mM range [58]. Inocula were suspended in sterile Biolog FF inoculating fluid and adjusted to a transmission of 81 and 62% at 590 nm for Myco and other micro-organisms, respectively. Plates were incubated in darkness at 25 °C, and growth (optical density) at 750 nm after 0, 12, 18, 24, 36, 42, 48, 60, 66, 72, 84, 90, 96, 114, and 138 h using an Enspire Multimode Reader (Perkin Elmer, Waltham, MA, USA) was monitored. The precise composition of all dual culture bioassay media was established in light of literature and confronted with the nutrient resources of plates.

### 5.5. Data Expression and Statistical Analysis

With the aim of considering complete growth and/or mycotoxin production kinetics, and not only a final point, the area under the growth/mycotoxin production curves were calculated for the antagonistic test and nutrient profiling test.

Statistical data analysis was performed with R Software (3.4.4, R Foundation for Statistical Computing, Vienna, Austria, 2017). Normality and homogeneity of variances were checked with the Shapiro–Wilk test (with Holm–Bonferroni correction) and Levene’s test, respectively. For each pathogen, the effect of BCA treatments was tested with a one-way ANOVA and multiple comparisons of means were done with Tukey’s test (α = 0.05). For the principal component analysis (PCA), data were standardized, and the analysis was done on the correlation matrix with the FactoMine R package. The first two components were retained in both cases. For the visualization of specific differences between microorganisms nutrient profiling, the negative control was subtracted and a heatmap was built with the ggplot package. The color scale indicates differential nutrient consumption in comparison with other micro-organisms (row comparison).

## Figures and Tables

**Table 1:** Selected media for dual culture bioassay. CM: common medium for all BCAs confrontation of concerned pathogen; SM: Specific media for each BCA-pathogen confrontation of concerned pathogen. Myco: Mycostop^®^, Xeda: Xedavir^®^, Poly: Polyversum^®^.

Selected Media for Dual Culture Bioassay (Pathogen–BCA)				
Pathogens	*F. Graminearum*		*F. Verticillioides*	
BCA	CM	SM	CM	SM
Myco	CYA	CZA	PDA	CYA
Xeda	CYA	CZA	PDA	MYA
Poly	CYA	OMA	PDA	OMA

**Table 2:** Contrasting characteristics of the selected commercial biocontrol agents.

BCAs, Commercial Products	Mycostop	Xedavir	Polyversum
Strain	*Streptomyces griseoviridis* strain K61	*Trichoderma asperellum* strain TV1	*Pythium oligandrum* strain M1/ATCC 38472
Family	Actinomycete	Ascomycete	Pythiaceae
Kingdom	Bacteria	Fungi	Oomycete
Plant/soil of isolation	*Sphagnum* peat	Root of tomato plant	Sugar beet
Locations of isolation	Finland	Italy	Czechoslovakia
Recommended Use	General soil treatment	General soil treatment	Barley and wheat aerial treatment
Target(s)	Soil-borne pathogen	Soil-borne pathogen	*Fusarium graminearum*
Commercialization company	Lallemand Plant Care^®^	Xeda International^®^	DeSangosse^®^
Date of marketing	2014	2013	2015
Identified general mechanism of action	Production of antimicrobial compound, mycoparasitism, plant defense induction	Mycoparasitism, plant defense induction	Mycoparasitism, plant defense induction
/on mycotoxigenic fungi	/none	/none	/on *F. graminearum*

**Table 3:** MS/MS parameters for isotope-labelled internal standard (IS). MRM: multiple reaction monitoring.

IS	Polarity	MRM	EC
DON C13	−	370.3 > 59.0	35
FB_1_ C13	+	756.3 > 356.5	−47

**Table 4:** MS/MS parameters for mycotoxins. Q: quantification; q: qualification.

Mycotoxin	Polarity	MRM Q	EC MRM Q	MRM Q	EC MRM Q	R^2^ Calibration Curve	IS
Fumonisin B_1_	+	722.35 > 334.5	−43	722.35 > 352.0	−44	0.9982	FB_1_ C13
Fumonisin B_2_	+	706.2 > 318.4	−37	706.2 > 3336.4	−44	0.9980	FB_1_ C13
DON	−	355.0 > 59.0	35	355.0 > 265.1	35	0.9998	DON C13
